# Two Distinct Endocrine Conditions in a Single Pediatric Patient: Congenital Adrenal Hyperplasia and Type 1 Diabetes Mellitus

**DOI:** 10.7759/cureus.100979

**Published:** 2026-01-07

**Authors:** Cindy Gomes, Mariana Bravo, Ariana Goncalves Marques, Joana Rosmaninho-Salgado, Alice Mirante

**Affiliations:** 1 Department of Paediatrics, Unidade Local de Saúde do Médio Tejo, Torres Novas, PRT; 2 Department of Paediatric Endocrinology, Hospital Pediátrico de Coimbra, Unidade Local de Saúde de Coimbra, Coimbra, PRT; 3 Department of Paediatrics, Unidade Local de Saúde da Região de Leiria, Leiria, PRT; 4 Department of Medical Genetics, Hospital Pediátrico de Coimbra, Unidade Local de Saúde de Coimbra, Coimbra, PRT

**Keywords:** 21-hydroxylase deficiency, congenital adrenal hyperplasia, cyp21a2 gene mutation, hyperandrogenism, type 1 diabetes mellitus

## Abstract

Non-classical congenital adrenal hyperplasia (NCCAH) is an autosomal recessive disorder most commonly caused by partial 21-hydroxylase deficiency, typically presenting with late-onset hyperandrogenism. Type 1 diabetes mellitus (T1DM) is an autoimmune disease characterized by the destruction of pancreatic beta cells, although its coexistence with NCCAH, as described in the literature, is rare.

We report an adolescent girl previously diagnosed with NCCAH at six years of age, identified due to premature adrenarche, elevated 17-hydroxyprogesterone and androstenedione, genetically confirmed by a homozygous *CYP21A2* c.1683G>T p.(Arg561Leu) pathogenic variant. During adolescence, she exhibited progression of hyperandrogenism, requiring glucocorticoid therapy. At 16 years of age, she presented to the emergency department with polydipsia, polyuria, weight loss, and recurrent fungal infections. Laboratory evaluation revealed hyperglycemia (435 mg/dL), a glycated hemoglobin (HbA1c) of 11.5%, without ketoacidosis, and positivity for anti-GAD65 and anti-islet cell antibodies, establishing the diagnosis of T1DM.

This case highlights important diagnostic and clinical considerations when hereditary endocrine diseases coexist with autoimmune diseases. Although NCAAH is not autoimmune, the location of *CYP21A2* within the major histocompatibility complex (MHC)--a genomic region rich in immunoregulatory genes--may contribute to a shared genetic susceptibility background in a subset of patients.The coexistence of NCCAH and T1DM is uncommon but may be partially explained by the location of the *CYP21A2* gene within the major histocompatibility complex (MHC), a genomic region associated with autoimmune susceptibility. Shared immunogenetic mechanisms may therefore contribute to the concurrent occurrence of these conditions, despite NCCAH not being classically autoimmune. This case highlights the importance of a thorough etiological evaluation in patients with hyperandrogenism, as well as the need for ongoing clinical follow-up, considering the possibility, albeit rare, of the development of other pathologies.

## Introduction

Non-classic congenital adrenal hyperplasia (NCCAH) is an autosomal recessive disorder caused by partial 21-hydroxylase deficiency, in which the enzyme retains some residual activity generally ranging from 20-70% [1-3].

Clinical manifestations depend on the degree of residual enzymatic activity, have a late onset, and result from an excess of circulating androgens. These may include premature pubarche, accelerated linear growth velocity, and advanced bone age [1,2]. Given the absence of clinical signs of adrenal insufficiency, establishing a differential diagnosis with other causes of hyperandrogenism is essential, which often makes this a particularly challenging diagnosis [1].

Type 1 diabetes mellitus (T1DM) is an autoimmune disease characterized by the destruction of pancreatic beta cells and the consequent absence of insulin production [2,4]. Children with T1DM are at increased risk of developing other autoimmune endocrinopathies, particularly autoimmune thyroiditis and primary adrenal insufficiency [4]. However, the association with NCCAH is rare and is scarcely described in the literature [2,4].

The molecular basis of NCCAH is complex due to the genomic organization of the RCCX module within the MCH class III region, where CYP21A2 lies adjacent to its highly homologous pseudogene CYP21A1P. This architecture predisposes to gene conversions and unequal crossing-over, the primary mechanisms underlying the >200 known pathogenic variants. The variant c.1683G>T p.(Arg561Leu) is one of the most frequent alleles associated with the non-classical form and is typically correlated with a milder biochemical phenotype.

Shared immunogenetic mechanisms between NCCAH and T1DM involve genetic variants associated with autoimmunity, particularly within the major histocompatibility complex, highlighting the HLA-DR3-DQ2 and HLA-DR4-DQ8 alleles, which increase the risk for T1DM as well as the autoimmune pathology of the adrenal gland [1,3-6]. Additionally, several genes (PTPN22, CTLA4, BACH2, IFIH1, and IL2RA) implicated in both conditions promote loss of immune tolerance and activation of autoreactive T lymphocytes [3-5,7].

Although the coexistence of NCCAH and T1DM in the pediatric population can be explained by immunogenetic mechanisms common to both conditions, cases reported in the literature remain rare [1-7].

Although NCCAH itself is not an autoimmune disease, the proximity of CYP21A2 to immunoregulatory loci within the MHC raises interest in whether some patients with structural variants or recombination events in this region may have increased susceptibility to autoimmunity. This does not imply causality, but may reflect shared genomic environments and linkage with HLA haplotypes associated with T1DM.

This report describes the case of a child initially diagnosed with NCCAH, who subsequently developed T1DM. This case underscores the importance of screening for other endocrine comorbidities in children and adolescents with a prior diagnosis of endocrine pathology, even in the presence of atypical symptomatology. The identification of multiple endocrinopathies in pediatric patients requires rigorous clinical surveillance and multidisciplinary follow-up, as it can significantly influence therapeutic management and prognosis.

## Case presentation

A female child was referred to the Paediatric Endocrinology Department due to suspected premature thelarche reported by the referring physician.

She was born following a well-monitored and uneventful pregnancy. Prenatal ultrasound examinations revealed no abnormalities. She was delivered vaginally at 40 weeks, with no need for resuscitation. Physical examination at birth revealed no abnormalities. There was no relevant past medical history, including no history of salt-wasting crisis. The parents were non-consanguineous. There was no relevant family history, namely, disorders of sex development, precocious puberty, menstrual irregularities, or infertility.

At the first Paediatric Endocrinology consultation, at six years of age, pubic hair and apocrine odour had been present for six months, and axillary hair for one month. Physical examination revealed sparse, lightly pigmented hair, chiefly along the medial border of the labia majora. There was no breast development or signs of oestrogenisation of the external genitalia. Laboratory evaluation demonstrated elevated 17-hydroxyprogesterone and androstenedione, while dehydroepiandrosterone sulphate (DHEA-S) was within the normal range. Serum cortisol, sodium, potassium, and testosterone levels, as well as adrenocorticotropic hormone (ACTH), were within reference ranges. Blood pressure was normal. Renal and adrenal ultrasonography showed no abnormalities. The laboratory results, along with their corresponding reference value, are presented in Table 1. Genetic analysis of the CYP21 gene identified a homozygous 1683G>T variant. Hydrocortisone was prescribed for stress dosing during intercurrent illness.

**Table 1:** Hormonal evaluation laboratory results with reference ranges DHEA-S: dehydroepiandrosterone sulphate; ACTH: adrenocorticotropic hormone

	6 years	12 years	16 years	Reference range
Height (Z-score)	+1.12 SD	-0.29 SD	-1.24 SD	Normal range: −2 to +2 SD
17-hydroxyprogesterone (ng/mL)	29	13	3.3	Pre-menarche: 0.1–1.7; Post-menarche: 0.31-2.41
Androstenedione (ng/mL)	1.1	-	1.5	6y: 0.04–0.42; 16y: 0.49-1.31
DHEA-S (µg/dL)	85.8	398	360	6y: 5-94; 12y: 9-170; 16y: 100-490
Cortisol (µg/dL)	10.6	9.6	12	6y: 5-25; 12 y: 5-25; 16y: 3.7-19.4
Sodium (mmol/L)	139	-	-	136-145
Potassium (mmol/L)	4,34	-	-	3.5-5.1
Total testosterone (ng/mL)	<20	0.5	0.6	6 y: <35; 12y: 0.15-0.52; 16y: 0.17-0.99
ACTH (pg/mL)	29.9	25	12	6y: <46; 12y: 9-52; 16y: 7.2-63.3

During follow-up, a slow and progressive increase in pubic and axillary hair was observed. Breast development began at eight years of age, and menarche occurred at nine years and seven months, consistent with early but not precocious puberty. There was no evidence of central precocious puberty, and no treatment targeting pubertal suppression was initiated. At 12 years of age, the patient reported progressive hirsutism. Laboratory evaluation revealed elevated 17-hydroxyprogesterone and DHEA-S levels. Pelvic ultrasonography demonstrated a pubertal uterus and ovaries of normal size and echostructure. Daily glucocorticoid therapy was initiated, resulting in a prompt decrease in the previously elevated hormone levels.

At 16 years of age, the medication was discontinued by the patient. At the last follow-up visit, at 16 years and 7 months of age, the patient reported no concerns, and laboratory evaluation limited to the hormonal axes showed an elevation of androstenedione and 17-hydroxyprogesterone. Growth remained within normal limits throughout follow-up. The laboratory results, along with their corresponding reference value, are presented in Table 1.

At 16 years and 9 months of age, she presented to the Emergency Department with polydipsia, polyuria, nocturia, a 7% weight loss, and recurrent vaginal fungal infections, with an evolution of approximately 3 months. At admission, capillary blood glucose was 435 mg/dL, and blood ketone levels were 1.2 mmol/L. Venous blood gas analysis showed no evidence of diabetic ketoacidosis. Laboratory evaluation revealed an elevated blood glucose level and increased glycated hemoglobin (HbA1c). Serum insulin and C-peptide levels were reduced but remained within the reference range. Antibodies against the islets of Langerhans and anti-GAD65 antibodies were positive. The patient did not present with obesity or clinical features of insulin resistance. Insulin therapy was initiated following diagnosis using a multiple daily injection regimen, with doses within the expected range for newly diagnosed type 1 diabetes. Hydrocortisone therapy was reassessed at the time of diabetes diagnosis and adjusted according to endocrine needs. The patient had previously been screened for autoimmune thyroid disease, with normal thyroid function tests and no evidence of autoimmune thyroiditis. There was also no clinical or laboratory evidence of anemia. Laboratory findings related to diabetes are presented in Table 2, in which reference values correspond to diagnostic thresholds rather than normal ranges.

**Table 2:** Laboratory evaluation at the time of the diagnosis of type 1 diabetes mellitus

	16 years	Reference Range for Alteration
pH	7.4	>7.3
HCO3 (mmol/L)	20.4	>18
Glucose (mg/dL)	435	>200
Glycated hemoglobin (%)	11.5	<6
Serum insulin (µIU/mL)	5.4	<30
C-peptide (ng/mL)	1.5	0.78–5.19

## Discussion

This case documents the rare coexistence of NCCAH and T1DM--two pathophysiologically distinct conditions: one hereditary and enzymatic, the other autoimmune. Their simultaneous occurrence raises clinically relevant questions regarding genetic background, phenotypic expression, and surveillance.

This clinical case describes an adolescent with non-classical congenital adrenal hyperplasia (NCAH) due to 21-hydroxylase deficiency who subsequently developed type 1 diabetes mellitus (T1DM). Although both conditions are relatively common when occurring independently, their concomitant manifestation is rare and has only been described in the literature as isolated case reports, representing the coexistence of a hereditary metabolic disorder and an autoimmune disease [2,8].

The patient carries the c.1683G>T p.(Arg561Leu) variant in homozygosity in the CYP21A2 gene. This missense variant is among the most common pathogenic alleles associated with the non-classical CAH. This variant represents a reduction in 21-hydroxylase activity, ranging from 20-80%, compatible with normal mineralocorticoid synthesis and largely preserved cortisol at baseline, but with insufficient reserve under physiologic stress or during puberty [1,9].

In agreement with published literature, the adolescent presented with premature adrenarche during childhood, accompanied by elevated androgen levels. During adolescence, she developed progressive hirsutism, as well as increased levels of 17-hydroxyprogesterone and DHEA-S, confirming that residual enzymatic activity may be insufficient to maintain hormonal balance during periods of increased adrenocortical stimulation, such as puberty [1,8].

While the association between autoimmune adrenal insufficiency (Addison’s disease) and T1DM is well-established in the context of autoimmune polyglandular syndromes, these mechanisms do not apply to NCAH, as it is not an immune-mediated disorder [9,10]. Nevertheless, there is no evidence of a causal relationship between NCAH and T1DM; their coexistence may be partially understood in the context of the genomic location of the CYP21A2 gene and through a shared genetic susceptibility background, rather than a direct causal relationship. This gene lies within the class II major histocompatibility complex (MHC), a highly polymorphic region that harbors multiple loci involved in immune regulation, including HLA-DRB1 and HLA-DQB1, which are implicated in susceptibility to T1DM.

The chromosomal region encompassing CYP21A2 is particularly complex because it lies in the RCCX module of chromosome 6p21.3, a region prone to recombination events and gene conversions between CYP21A2 and its pseudogene CYP21A1P. The region neighbours loci such as TNXB, C4A/C4B, and, more broadly, class II HLA genes--major determinants of autoimmunity risk. Structural variants or recombination patterns in the RCCX region could be in linkage disequilibrium with HLA haplotypes associated with T1DM. Some CYP21A2 genotypes may segregate more frequently within families carrying T1DM-associated HLA-DR3/DQ2 or DR4/DQ8 alleles, without causation but reflecting shared inherited genomic backgrounds. No evidence supports that CYP21A2 variants directly increase autoimmune risk, but the genomic neighborhood may partially explain rare co-occurrences such as this case. Importantly, there is no evidence that CYP21A2 variants directly increase autoimmune risk. However, the complex genomic architecture of the MHC may partly explain rare co-occurrences such as the one described in this case. Additionally, subtle fluctuations in the hypothalamic-pituitary-adrenal axis, typical of NCAH, may modulate immune responses over time and potentially play a role in the development of autoimmune conditions in genetically predisposed individuals [1,9-11].

Although all reported mutations lead to 21-hydroxylase deficiency, the specific variants described in the literature differ from those identified in this case, highlighting the genetic heterogeneity and potential variability in clinical presentation [2,9].

This case highlights the importance of genetic testing in the differential diagnosis of hyperandrogenism, particularly when clinical features overlap with conditions such as polycystic ovary syndrome or other causes of peripheral androgen excess. Furthermore, it reinforces the need for long-term, multidisciplinary follow-up in patients with NCAH. It is essential to acknowledge the possibility, albeit rare, of the development of autoimmune diseases, especially in individuals carrying genetic variants located in critical regions of immune regulation.

## Conclusions

This case demonstrates a rare association between non-classical congenital adrenal hyperplasia and autoimmune type 1 diabetes mellitus, emphasizing the importance of an accurate etiological diagnosis in complex clinical contexts. Although the two diseases arise from distinct mechanisms--one enzymatic and hereditary, the other autoimmune--the genomic location of CYP21A2 within the MHC raises the possibility of shared susceptibility environments in select patients. The coexistence of a hereditary endocrine disorder and a common autoimmune disease illustrates how multiple hormonal alterations may interact and modify the clinical profile, necessitating an integrated and personalized approach.

The well-documented clinical presentation, longitudinal follow-up, and genetic confirmation make this case particularly significant. It underscores the need for further investigation into the interactions between genetic alterations within the MHC region and the risk of autoimmune diseases in patients with NCAH. Future studies may clarify whether a subgroup of patients presents an increased predisposition to autoimmune conditions, thereby contributing to the development of more appropriate surveillance strategies.
